# Proteome-wide Mendelian randomization and colocalization analyses identify potential biomarkers for schizophrenia

**DOI:** 10.3389/fpsyt.2026.1724567

**Published:** 2026-02-27

**Authors:** Jingyu Lin, Haiming Huang, Lin Chen, Yanyan Wei, Tianmei Si, Yunai Su, Yajuan Niu, Jingxu Chen

**Affiliations:** 1 Beijing Huilongguan Hospital, Capital Medical University, Beijing, China; 2 Department of Clinical Laboratory, Peking University First Hospital, Beijing, China; 3 Peking University Sixth Hospital, Peking University Institute of Mental Health, National Clinical Research Center for Mental Disorders (Peking University Sixth Hospital), NHC Key Laboratory of Mental Health (Peking University), Beijing, China

**Keywords:** biomarkers, colocalization, CTSS, Mendelian randomization, schizophrenia

## Abstract

**Background:**

We performed proteome-wide Mendelian randomization (MR) and colocalization analyses to explore the causal relationships between proteins and schizophrenia (SCZ).

**Methods:**

In the primary analysis, genetic instruments of 4,907 plasma protein from 35,559 Icelanders served as the exposure, summary statistics for SCZ (35,476 cases, 46,839 controls) Working Group of the Psychiatric Genomics Consortium (PGC) served as the outcome. The initial findings underwent sensitivity analyses and were externally validated using cis-pQTLs from the Fenland study (4,979 proteins, 10,708 participants) and UK Biobank Pharma Proteomics Project (UKB-PPP, 2923 proteins, 33,043 participants), and brain cis-eQTLs from Genotype-Tissue Expression (GTEx). Bayesian colocalization assessed shared causal variants. Protein-protein interactions with antipsychotic drug targets were explored.

**Results:**

In the primary analysis, genetically predicted levels of seven plasma proteins were significantly associated with SCZ risk: ADAM22 (OR = 0.85, *P* = 8.97×10^−7^), LIMA1 (OR = 0.43, *P* = 1.28×10^−5^), CTSS (OR = 1.27, *P* = 9.18×10^−7^), FOXO3 (OR = 2.80, *P* = 6.05×10^−6^), IRF3 (OR = 2.09, *P* = 1.15×10^−6^), KLC1 (OR = 2.17, *P* = 3.38×10^−^¹¹), and MMP16 (OR = 2.00, *P* = 1.60×10^−5^). External validation partially confirmed these associations: LIMA1, CTSS, FOXO3, KLC1, and MMP16 replicated in the Fenland study; ADAM22 and CTSS replicated in UKB-PPP; MMP16 and CTSS expression in specific brain tissues replicated using GTEx brain eQTLs. Colocalization strongly supported shared causal variants for FOXO3 (PPH4 = 0.966), IRF3 (PPH4 = 0.932), and LIMA1 (PPH4 = 0.985) with SCZ. CTSS, FOXO3, IRF3, and MMP16 showed interactions with known antipsychotic drug targets.

**Conclusion:**

This large-scale MR study provides robust evidence supporting causal roles for specific plasma proteins in SCZ pathogenesis, highlighting promising candidates for mechanistic studies and therapeutic development.

## 1 Background

Schizophrenia (SCZ) is characterized by the manifestation of psychotic symptoms and frequently associated with a decrease in social and occupational functioning, continues to present challenges in terms of its causes and treatment strategies (1). The etiology of SCZ is potentially attributable to alterations in cerebral architecture and functionality, coupled with neurotransmitter dysregulation, aberrant developmental processes, and genetic underpinnings (1). SCZ, a complex psychiatric disorder, the heritability of SCZ is estimated to range between 64% and 81% (2). In a largest genome-wide association study (GWAS) involving 76,755 individuals diagnosed with SCZ and 243,649 control individuals, the study delineates common variant associations across 287 distinct genomic loci (3). Notably, these associations predominantly converge on genes that are actively expressed in both excitatory and inhibitory neurons within the central nervous system, rather than in other tissues or cell types, underscoring the specific genetic underpinnings of SCZ (3). The identified associations also underscore critical processes intrinsic to neuronal functionality, encompassing synaptic organization, differentiation, and transmission (3). However, approximately 90% of the single-nucleotide polymorphisms (SNPs) identified by GWAS studies were located in a noncoding region (4).

Proteomic investigations not only enhance our molecular comprehension but also contribute to the identification of potential therapeutic targets, this is attributed to the pivotal role circulating proteins play in regulating molecular pathways (5). Human proteins are integral to numerous biological functions and predominantly serve as targets for pharmacological interventions (6). A previous study has elucidated that drug targets underpinned by genetic associations with diseases, exhibit a twofold increase in the probability of attaining market authorization (7). Prior studies have elucidated a multitude of potential biomarkers implicated in SCZ, such as inflammatory and oxidative stress markers (8). Nonetheless, to date, there remains an absence of protein-based markers that can be employed for the diagnostic and individualized treatment of SCZ. A potential explanation for the paucity of reliable biomarkers for SCZ may lie in the predominant reliance on cross-sectional study designs within this field. Such methodologies are inherently limited in their capacity to elucidate causal linkages between protein levels and SCZ, thereby engendering a landscape characterized by inconsistent findings.

Mendelian randomization (MR) constitutes a methodological approach employing genetic instrumental variable analysis, typically leveraging SNPs derived from GWAS. This technique is instrumental in inferring the causal influence of an exposure on an outcome through the utilization of genetic instruments. In this study, we aimed to explore the associations between plasma proteins and SCZ using MR analysis. First, MR was employed to elucidate potential causal relationships between plasma proteins and SCZ. The plasma proteomics data were derived from the deCODE Health study (9), and the outcome GWAS data were provided by the Working Group of the Psychiatric Genomics Consortium (PGC) (10). Second, the initial findings were rigorously validated by several methods to confirm their robustness, including Steiger filtering, MR-Egger regression, leave one out test, bidirectional MR causality detection, colocalization analysis, and phenotypic scanning. Third, we utilized three independent validation cohorts—including pQTL data from the Fenland Study and UK Biobank Pharma Proteomics Project (UKB-PPP), alongside brain eQTL data from the Genotype-Tissue Expression (GTEx) project, to rigorously assess the reliability of primary causal associations. Finally, we systematically mapped interaction networks among identified proteins and established molecular targets of current schizophrenia medications.

## 2 Methods

### 2.1 Selection of the instrumental variables

In this study, only IVs satisfying the following criteria were included: (1) showed genome-wide significant association (*P* ≤ 5 × 10 ^−8^) with the levels of plasma proteins; (2) were not situated inside the MHC (major histocompatibility complex) region; (3) SNPs found within 1 Mb of the protein-encoding gene were referred to as cis-SNPs; (4) had a minor allele frequency threshold of 0.01 was permitted for palindromic SNPs; (5) showed independent association: linkage disequilibrium (LD) r^2^ < 0.001 based on the 1000 Genomes European Sample Project (11); (6) had a smaller bias as all SNPs had F-statistic values of at least 10.

For the primary analysis, the plasma protein quantitative trait loci (pQTL) data were obtained from the deCODE Health study (9). The deCODE Health study conducted proteomic profiling on blood plasma samples from 35,559 Icelanders using the SomaScan platform and collected data on 4,907 proteins (9), index cis-SNPs for 1603 plasma proteins were included in the primary analysis.

For the external validation, the UKB-PPP data were used (12), 2,923 plasma proteins were measured among 33,043 UKB Europeans using Olink platform. Moreover, the Fenland study measured 4979 plasma proteins using SOMAscan assay in 10,708 participants were also included to replicate the primary results. The identified proteins from the primary analysis were included in the external validation using the same criteria.

### 2.2 Expression quantitative trait loci data of GTEx brain tissues

We utilized the *MRInstruments* R package to download the GTEx eQTL data (13). Focusing on psychiatric disorders, we specifically extracted brain eQTLs from the GTEx dataset, identifying 35,673 conditionally independent cis-SNPs linked to gene expression. In line with previous studies (14, 15), we selected genetic variants with significant associations to gene expression (eQTL *P* < 1 × 10^-4^) for further analysis.

### 2.3 GWAS summary statistics of SCZ

To minimize the bias associated with population heterogeneity, only aggregated data from the European cohort were utilized in this analysis. In the primary, the summary statistics of SCZ were obtained from the PGC, which included 35,476 SCZ cases and 46,839 controls of European ancestry (10).

### 2.4 MR analyses

In this study, we performed MR using *TwoSampleMR* (16) R package with the cis-pQTL of the plasma proteins as the exposure and SCZ as the outcome. To investigate the causal relationship between plasma proteins and SCZ, we employed inverse variance weighted (IVW) and the Wald ratio. In the primary analysis, Bonferroni correction was applied to adjust for multiple testing, and results were prioritized for further analysis based on a threshold *P*-value of 3.11 × 10 ^-5^ (0.05/1603 proteins).

### 2.5 Sensitivity analyses

We validated the significant MR results through sensitivity analyses as follows: (1) We conducted a leave-one-out analysis to determine whether the causal association was influenced by any single SNP, identifying outliers with a *P*-value of <0.05. (2) We performed MR-Egger regression to assess potential directional pleiotropy bias. The intercept from the Egger regression represents the average pleiotropic effect of all genetic variants. A value significantly different from zero (*P* < 0.05) indicates evidence of pleiotropy. (3) To ensure the correct causal direction between gene expression and schizophrenia risk, we performed Steiger filtering to test the directionality of putative causal associations. (4) Phenotype scanning: The SNPs were regarded as pleiotropic if they were associated with a recognized risk factors for SCZ, including education (17), cannabis use (1, 17), childhood adversity (1, 17) and drinking (18). We assessed pleiotropy of identified pQTLs using the ‘LD trait’ resource (https://ldlink.nih.gov/?tab=ldtrait) to query associations with other traits in prior GWAS.

### 2.6 Bidirectional MR causality detection

We also used bidirectional MR analysis to detect potential reverse causality, genetic instruments for SCZ were selected from the GWAS of PGC as the exposure, and pQTLs of the identified plasma proteins from the primary analysis as the outcome. The results were considered statistically significant at *P* < 0.05.

### 2.7 Bayesian colocalization analyses

The likelihood that two traits share the same causal variant was evaluated using Bayesian co-localization analyses. The analyses were performed using the *coloc* package R package (19). In this study, we established the prior probabilities as follows: for a SNP to be associated exclusively with trait 1 (p1), the probability was set at 1 × 10^-4^; for association solely with trait 2 (p2), the probability was similarly set at 1 × 10^-4^; and for the SNP’s association with both traits (p12, two traits sharing the same variant), the probability was established at 1 × 10^-5^. In current colocalization analyses, variants within ±500 kb of the lead SNP were included. Two traits were considered to have strong supporting evidence for colocalization if the posterior probability of sharing causal variants reached or exceeded 0.8.

### 2.8 Potential SCZ biomarkers and current antipsychotic medications targets interaction network

We constructed a protein-protein interaction (PPI) network for proteins suggestively associated with SCZ risk using and current antipsychotic medications targets. Current 15 antipsychotic medications and their molecular targets were curated from DrugBank. PPI networks were generated using STRING v12.0 (https://string-db.org).

## 3 Results

### 3.1 Screening causal proteins for SCZ in the primary analysis

**Figure 1:**
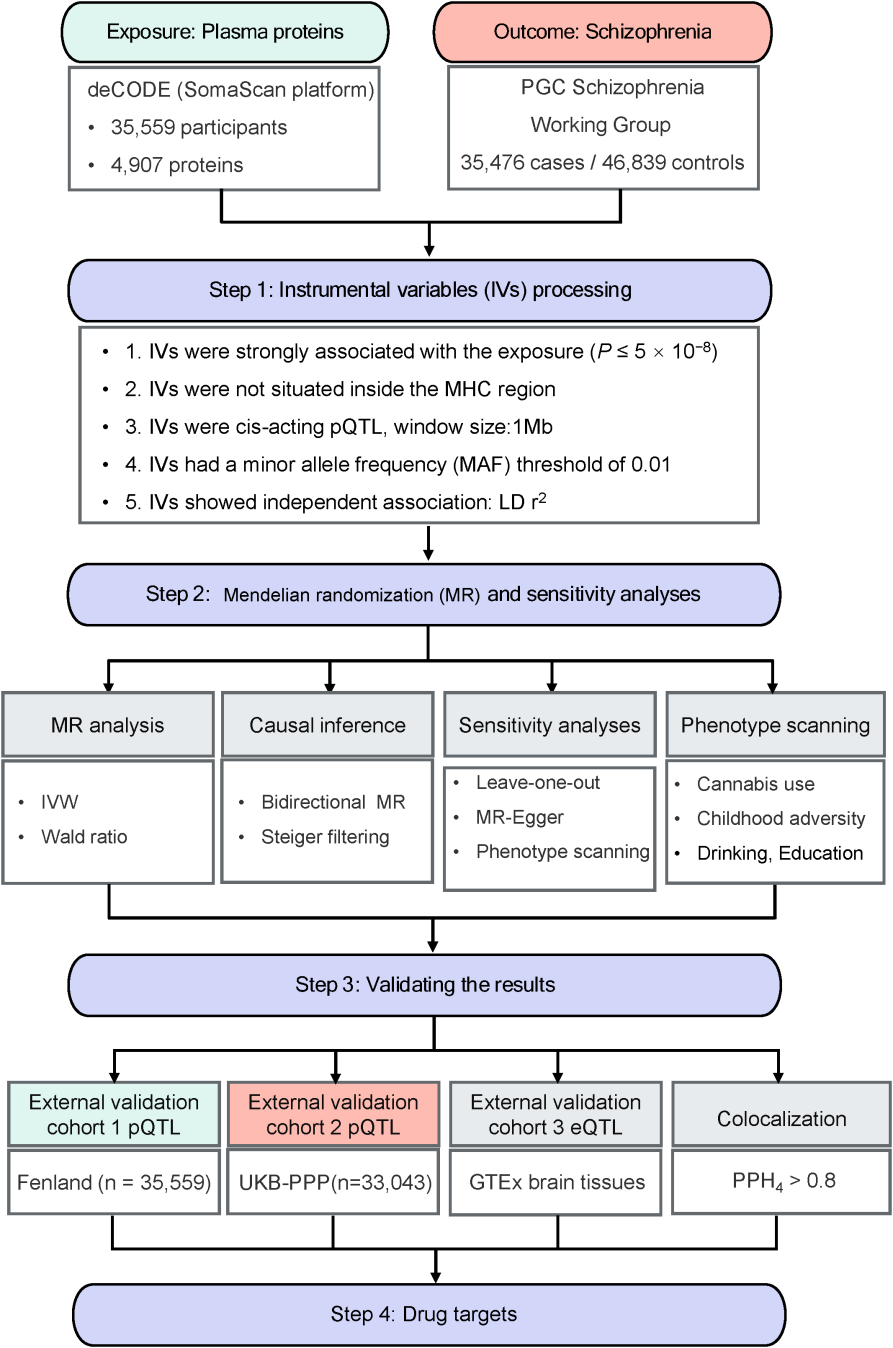
Study design for identification of potential biomarkers for causally associated with schizophrenia.

An overview of this study design was presented in **Figure 1**. The summary-level GWAS data of plasma proteins and SCZ was listed in **Supplementary Table S1**. Following thorough processing of plasma proteins, this study included 1,603 proteins in the primary analysis, the genetic instruments utilized have a minimal *F* statistic over 29.7, reducing the risk of weak instrument bias.

**Figure 2:**
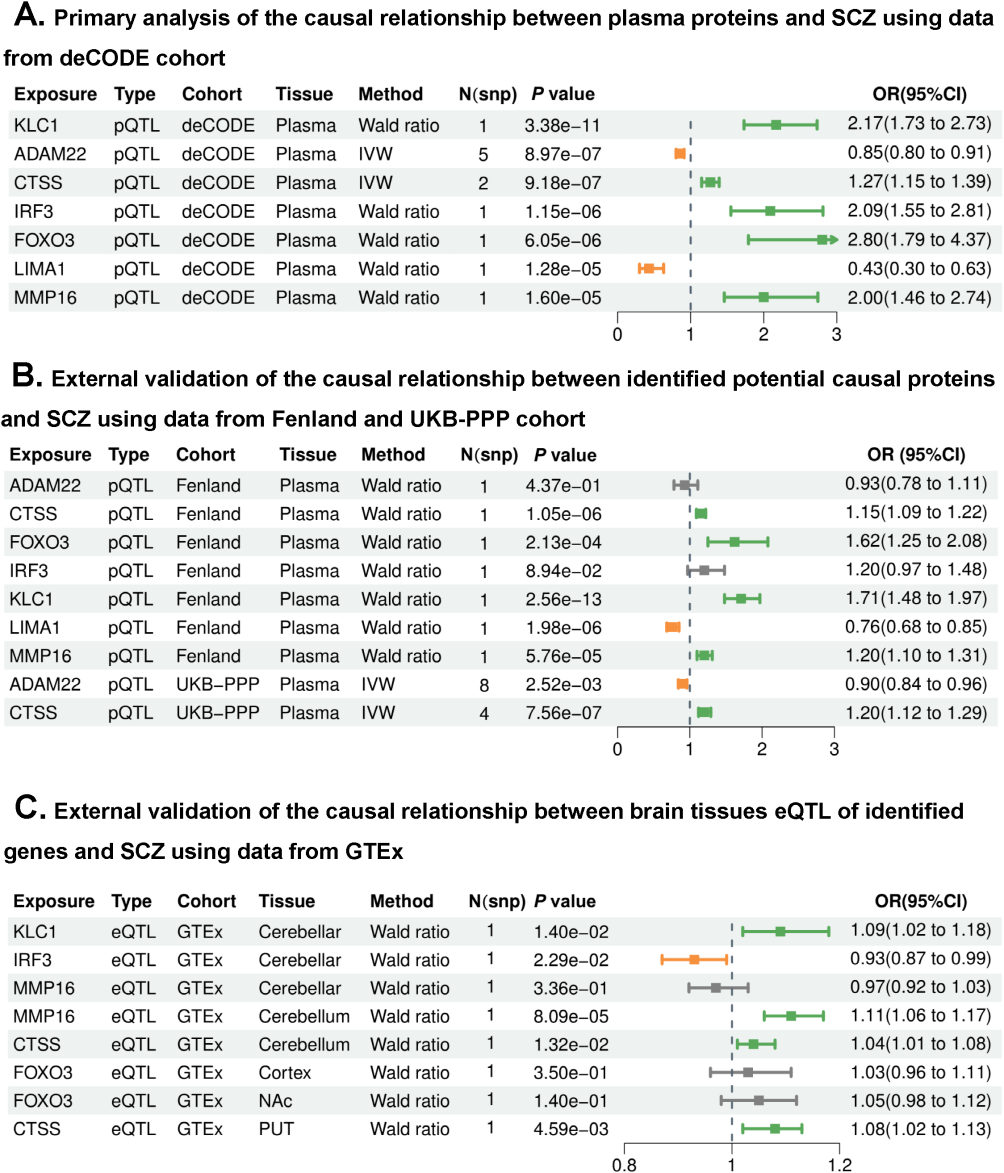
Mendelian randomization (MR) analyses identifying and validating potential biomarkers associated with schizophrenia risk. **(A)** Primary MR analysis of plasma proteins associated with schizophrenia risk using deCODE data. Odds ratios (OR) represent risk per standard deviation (SD) increase in genetically predicted protein levels, seven proteins were significantly associated with schizophrenia (*P* < 3.11 × 10^-5^). **(B)** External validation of the primary findings using cis-pQTLs from Fenland and UK Biobank Pharma Proteomics Project (UKB-PPP). **(C)** External validation of the primary findings using cis-eQTLs from GTEx.

After Bonferroni correction (*P* < 3.11 × 10^-5^, 0.05/1,603), MR analysis genetically predicted levels of seven proteins were significantly associated with SCZ risk, including ADAM metallopeptidase domain 22 (ADAM22), cathepsin S (CTSS), Forkhead Box O3 (FOXO3), Interferon Regulatory Factor 3 (IRF3), kinesin light chain 1 (KLC1), LIM domain and actin binding 1 (LIMA1) and matrix metallopeptidase 16 (MMP16). Specifically, decreased levels of LIMA1 (OR per SD, OR = 0.43, *P* = 1.28 × 10^-5^) and ADAM22 (OR = 0.85, *P* = 8.97 × 10^-7^), and increased levels of FOXO3 (OR = 2.80, *P* = 6.05 × 10^-6^), KLC1 (OR = 2.17, *P* = 3.38 × 10^-11^), IRF3 (OR = 2.09, *P* = 1.15 × 10^-6^), MMP16 (OR = 2.00, *P* = 1.60 × 10^-5^) and CTSS (OR = 1.27, *P* = 9.18 × 10^-7^) were associated with increased risk of SCZ (**Figure 2A**, **Supplementary Table S2**).

### 3.2 Sensitivity analyses of the relationship between the seven identified proteins and SCZ

First, bidirectional MR analyses revealed no causal effect of SCZ on the levels of the seven identified proteins (**Supplementary Table S3**). Second, Steiger filtering confirmed the directional consistency of our findings (**Supplementary Table S4**). Third, the leave-one-out analysis detected no single SNP significantly altering the causal association (**Supplementary Table S5**), and MR-Egger regression detected no directional pleiotropy bias (**Supplementary Table S6**). Fourth, SNPs associated with each candidate protein were queried in the LD trait database, no SNPs were associated with known SCZ confounders (**Supplementary Table S7**).

### 3.3 External validation of the relationship between plasma proteins and SCZ

To replicate the primary findings, we used cis-pQTLs from the Fenland study as the exposure (**Figure 2B**, **Supplementary Table S8**), SCZ from PGC as the outcome, decreased level of LIMA1 (OR = 0.76, *P* = 1.98 × 10^-6^) was still associated with increased risk of SCZ, whereas increased levels of CTSS (OR = 1.15, *P* = 1.05 × 10^-6^), FOXO3 (OR = 1.62, *P* = 2.13 × 10^-4^), KLC1 (OR = 1.71, *P* = 2.56 × 10^-13^) and MMP16 (OR = 1.20, *P* = 5.76 × 10^-5^) were associated with increased risk of SCZ. However, levels of ADAM22 and IRF3 were not associated with the risk of SCZ.

Furthermore, we used cis-pQTLs from the UKB-PPP as the exposure (**Figure 2B**, **Supplementary Table S8**), SCZ from PGC as the outcome, we found decreased level of ADAM22 (OR = 0.90, *P* = 0.003) and increased level of CTSS (OR = 1.20, *P* = 7.56 × 10^-7^) were also associated with increased risk of SCZ. The remaining five proteins were not identified in the processed UKB-PPP dataset.

Finally, we used cis-eQTLs from GTEx brain tissues as the exposure (**Figure 2C**, **Supplementary Table S8**), SCZ from PGC as the outcome. We identified eight cis-eQTLs for CTSS, MMP16, IRF3, KLC1, and FOXO3 to serve as the instrumental variables for MR validation, however, eQTL SNPs for ADAM22 and LIMA1 were not found were excluded. At Bonferroni significance (*P* = 0.006, 0.05/8), only increased level of MMP16 in the cerebellum (OR = 1.11, *P* = 8.09 × 10^-5^) and increased level of CTSS in the putamen basal ganglia (OR = 1.07, *P* = 0.005) associated with increased risk of SCZ. Moreover, levels of KLC1 and IRF3 in the cerebellar hemisphere, and CTSS in the cerebellum tend to be associated with the risk of SCZ.

### 3.4 Colocalization analyses

**Figure 3:**
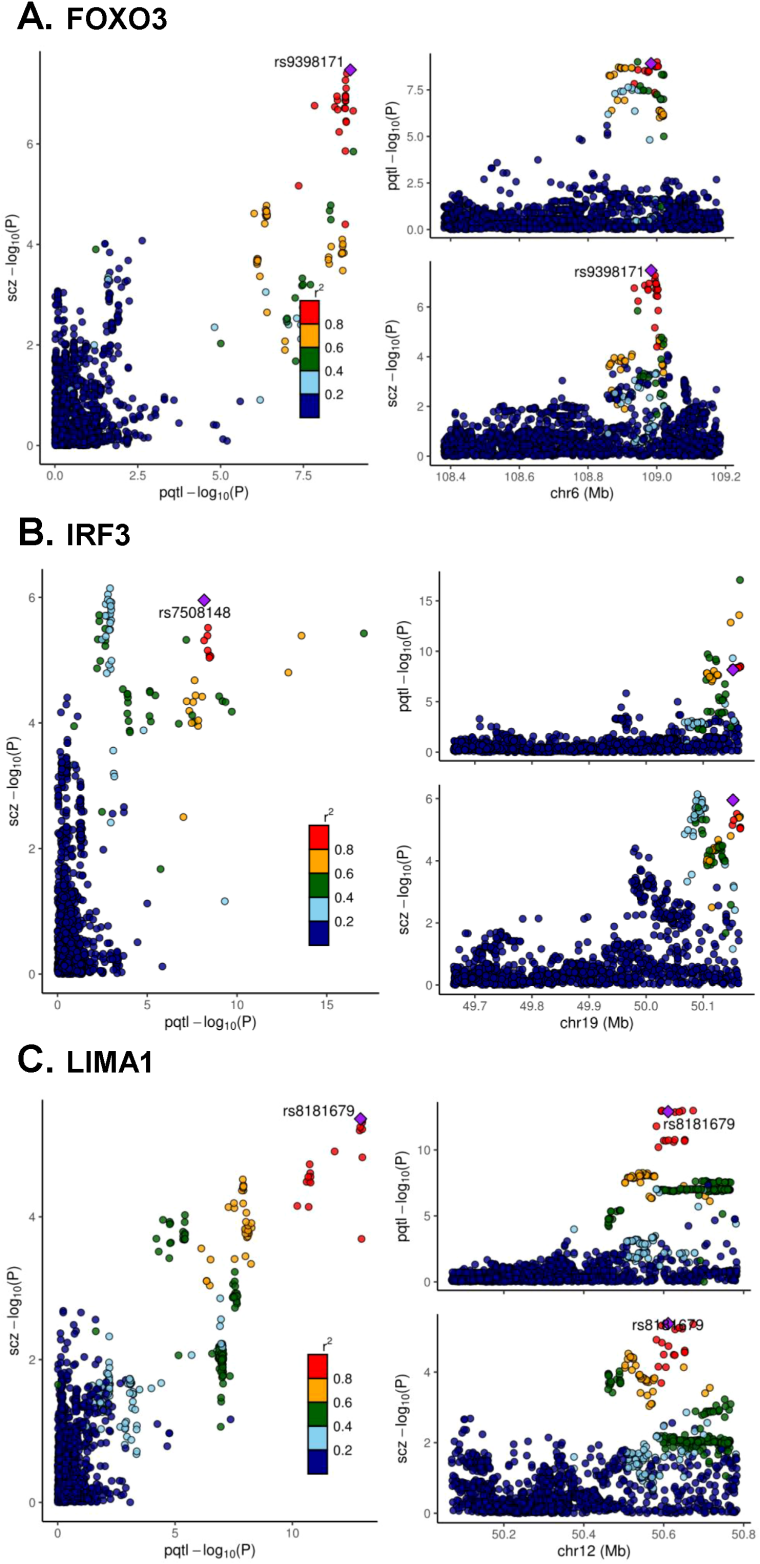
Colocalization analyses of cis-pQTL for FOXO3 **(A)**, IRF3 **(B)** and LIMA1 **(C)** with schizophrenia.

Colocalization analysis (posterior probability > 0.8) provided evidence of a shared causal variant underlying the associations of seven blood markers with SCZ. Bayesian co-localization strongly suggested that FOXO3 (coloc.abf-PPH4 = 0.966) (**Figure 3A**), IRF3 (coloc.abf-PPH4 = 0.932) (**Figure 3B**) and LIMA1 (coloc.abf-PPH4 = 0.985) (**Figure 3C**) shared the same variant with SCZ (**Supplementary Table S9**), respectively.

### 3.5 Association of potential drug targets with current antipsychotic drugs

**Figure 4:**
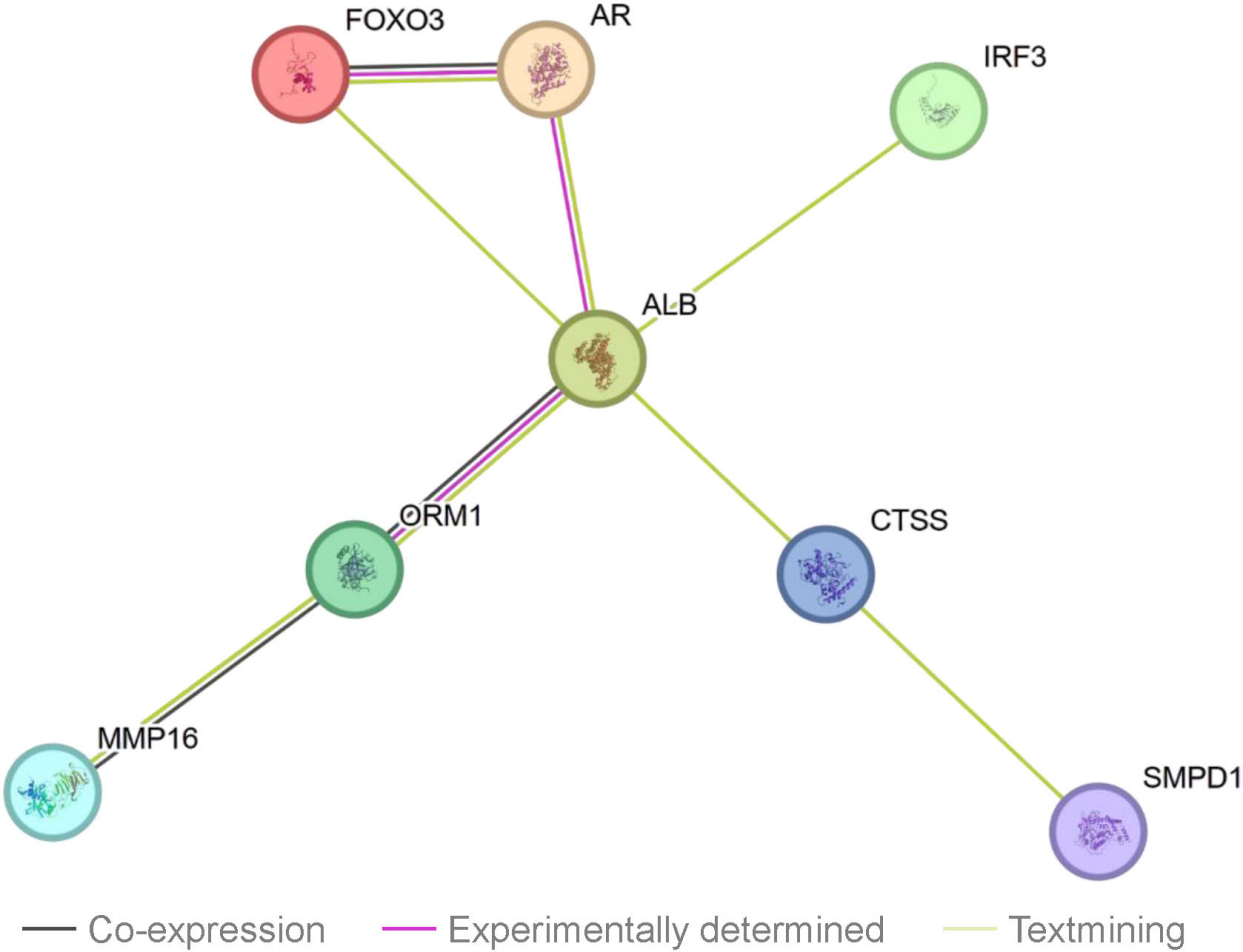
Protein-Protein interaction (PPI) network between current antipsychotic medications targets and identified potential drug targets.

Using the STRING protein-protein interaction (PPI) network, we investigated interactions between seven Mendelian randomization-identified proteins (CTSS, LIMA1, IRF3, MMP16, ADAM22, FOXO3 and KLC1) and established antipsychotic drug targets from DrugBank (**Supplementary Table S10**). Four proteins demonstrated interactions with known antipsychotic drugs targets (**Figure 4**). Notably, all four schizophrenia-associated proteins (CTSS, IRF3, FOXO3 and MMP16) exhibited direct or indirect connections to the drug target albumin (ALB). ALB is a known target of established antipsychotic medications, including olanzapine, chlorpromazine, and aripiprazole. Furthermore, FOXO3-AR was determined to have the most reliable interactions (known interactions). Specifically, FOXO3 was associated with the androgen receptor (AR), the target of fluphenazine.

## 4 Discussion

In this study, we employed a comprehensive MR framework, integrating large-scale plasma pQTL and brain tissues pQTL datasets, to identify novel proteins causally associated with SCZ. Our initial discovery using deCODE data identified seven proteins (ADAM22, CTSS, FOXO3, IRF3, KLC1, LIMA1, MMP16) associated with SCZ risk. We further validated the schizophrenia associations of these seven proteins through plasma pQTLs (UKB-PPP, Fenland), brain eQTLs (GTEx), and colocalization analysis.

In this study, we used three pQTL datasets from deCODE, Fenland, and UKB-PPP, along with brain tissue eQTL data from GTEx, consistently support the association between increased level of CTSS and increased risk of SCZ. CTSS is a unique cysteine protease implicated in various diseases, including chronic obstructive pulmonary disease (COPD), pain, cancer, diabetes, obesity, autoimmune disease, and cardiovascular disease (20). In the nervous system, overexpression of CTSS in hippocampal neurons of young mice impairs spatial learning and memory behaviors (21), while knocking down CTSS in hippocampal neurons reverses spatial learning and memory deficits in aged mice (21). The underlying mechanism may involve dysfunction of neuroinflammation pathways, as CTSS overexpression induces an inflammatory microenvironment in hippocampal neurons and activates microglia towards an M1 pro-inflammatory phenotype, while also elevating levels of various pro-inflammatory markers (21). Furthermore, another animal study found that downregulate CTSS expression leads to the liberation of olfactory receptor transcription factor-1 from CREB-binding protein (CBP), enabling it to bind to the promoter region of BDNF (brain-derived neurotrophic factor) and subsequently activate BDNF transcription. Furthermore, a recent study discovered that macrophage-derived CTSS drives age-dependent disruption of the blood-CSF barrier (22). Additionally, CTSS has been linked to dysregulation of the tryptophan metabolic pathway in ulcerative colitis (23). Therefore, CTSS may contribute to the pathogenesis of schizophrenia by mediating neuroinflammation, learning and memory processes, blood-CSF barrier integrity, tryptophan metabolism, and BDNF signaling pathways. However, the precise mechanisms warrant further investigation.

Previous studies have found that FOXO3 is associated with autism spectrum disorder (ASD) and SCZ, representing a shared biological underpinning for both disorders (24). Another study also identified FOXO3 (rs10457180) as one of shared genetic loci between SCZ and intracranial volume (25). Current evidence indicates that FOXO3 is involved in neurodevelopment, cellular homeostasis, cell death, inflammation, metabolism, and stress adaptation (26–29). It is worth noting that genetic variation in the FOXO3 gene is strongly associated with human longevity (30). FOXO3 is highly expressed in the brain (27), where it regulates neural stem cell homeostasis and neurogenesis (29, 30). FOXO proteins also control dendrite and spine development, and influence the integration of newly generated dentate granule neurons into hippocampal circuitry (26).

ADAM22 plays a significant role in synaptic function and neural development. Substantial evidence indicates a close association between ADAM22 and epilepsy (31–33). ADAM22 forms a synaptic complex with LGI1 and PSD-95, serving as a crucial component for synaptic connectivity and signal transmission (33). Furthermore, ADAM22 is associated with neuronal development and axonal guidance (33). Hence, ADAM22 may contribute to the pathogenesis of schizophrenia by mediating synaptic transmission and neural development.

In this study, our study revealed a tissue-specific divergence in the direction of effect for IRF3: elevated plasma protein levels were associated with increased schizophrenia risk, whereas higher cerebellar mRNA expression showed a protective effect. This discrepancy likely reflects the compartment-dependent roles of IRF3 in schizophrenia pathology. In the CNS, IRF3 is essential for maintaining synaptic homeostasis and the excitation/inhibition (E/I) balance. Reduced brain IRF3 activity has been linked to hyper-glutamatergic transmission and behavioral deficits in animal models (34), suggesting that higher central expression is neuroprotective. Conversely, in the peripheral circulation, IRF3 acts as a master regulator of Type I interferon signaling. Chronic systemic upregulation may drive a pro-inflammatory state that compromises blood-brain barrier integrity and exacerbates neuroinflammation (35, 36). Thus, our findings support a model where schizophrenia risk is modulated by a trade-off between defective central synaptic regulation and excessive peripheral immune activation. IRF3 is an interferon-responsive transcription factor and serves as a key transcription factor for interferon-stimulated genes (ISGs), which play roles in antiviral defense and immune response regulation (37). IRF3 plays a pivotal role in neuroinflammation; its excessive activation can drive the polarization of microglia towards a pro-inflammatory M1 phenotype, leading to the release of pro-inflammatory cytokines and chemokines (37). IRF3 is also involved in the endoplasmic reticulum (ER) stress response, helping cells cope with stress by regulating the expression of ER stress-related genes (38).

A previous study suggested strong colocalization evidence between KLC1 and schizophrenia, indicating that this gene shares a common genetic basis with schizophrenia risk loci (39). This finding aligns with results from an epigenome-wide association study, which also suggested that DNA methylation at this locus may play a role in schizophrenia risk (40). KLC1 is a microtubule-based motor protein that plays a significant role within cells, responsible for the positioning and transport of various biomolecules (41). Kinesin-1 is the most abundant motor protein family in the brain and is involved in neuronal differentiation processes (41). Hi-C interactions within the KLC1 promoter region further support the putative regulatory mechanism (42).

LIMA1 is a critical regulator mediating intestinal cholesterol absorption (43) However, the role of LIMA1 in psychiatric or neurological disorders remains unclear. For MMP16, large-scale GWAS studies have identified rs7004633 (near the MMP16 gene) as a SNP exhibiting genome-wide significant association with schizophrenia (44). An integrative analysis also implicates MMP16 as a causative gene for schizophrenia (45). A small-scale study suggested an association between the rs7004633 (MMP16) SNP and the Cognitive-Perceptual features of schizophrenia. MMP16 is an enzyme responsible for degrading the extracellular matrix. MMP16, along with other genes in its family, exhibits unique spatiotemporal expression patterns in the developing and adult human brain, with significantly higher expression levels in neurons compared to oligodendrocytes and microglia (45). In a rat model of hypoxia, MMP16 expression was significantly up-regulated in the prefrontal cortex, striatum, and hippocampus on postnatal day 1, suggesting an important role for this gene in neurodevelopment (46).

Despite leveraging the robust MR framework and employing multiple validation strategies to investigate the causal relationship between plasma proteins and SCZ our study is subject to several limitations that warrant consideration. First, the primary findings of this study are derived from plasma proteins. Causal effects observed for proteins in plasma may not directly reflect biological mechanisms within the brain or fully capture brain-specific protein alterations. Future research should integrate proteomic data from cerebrospinal fluid (CSF) or brain tissue itself to provide more direct evidence. Second, although this study identified several novel proteins significantly associated with schizophrenia, their mechanisms of action remain poorly understood. Future investigations, including animal studies or biomarker research, are warranted to explore the roles of these proteins in the nervous system and schizophrenia pathogenesis. Third, all GWAS data utilized in this study originated from individuals of European ancestry. Consequently, the results cannot be directly generalized to other ethnic populations. Future research needs to replicate these findings in multi-ethnic cohorts to evaluate the generalizability of the discoveries. Fourth, this study employed only cis-acting SNPs as genetic instruments for both pQTL and eQTL analyses. While cis-pQTLs are generally less susceptible to horizontal pleiotropy, relying solely on cis-acting instruments may limit the comprehensive exploration of complex causal relationships. Furthermore, when the number of genetic instruments was limited (particularly when only a single SNP was available), more robust sensitivity analyses could not be effectively implemented, potentially compromising the robustness of the causal inference.

## 5 Conclusions

This large-scale, multi-cohort Mendelian randomization study robustly identified seven plasma proteins with genetically predicted levels causally associated with SCZ risk. Deeper understanding of the biological functions and therapeutic relevance of these candidate proteins in SCZ requires future investigation.

## Data Availability

The original contributions presented in the study are included in the article/**Supplementary Material**. Further inquiries can be directed to the corresponding authors.
