# Supplementary material for: Proteome-wide Mendelian randomization and colocalization analyses identify potential biomarkers for schizophrenia
Source: Front Psychiatry. 2026 Feb 27;17:1724567. doi: 10.3389/fpsyt.2026.1724567 (PMC12982484; doi:10.3389/fpsyt.2026.1724567)
Supplement: Supplementary file 2 [file Table2.docx]

**Supplementary Materials For**

**Proteome-wide mendelian randomization and colocalization analyses identify potential biomarkers for schizophrenia**

**1. Calculation of F-statistic**

To assess the strength of our genetic instruments and avoid weak instrument bias, we calculated the F-statistic for each SNP using the following parameters: beta coefficient (β), standard error (*SE*), sample size (*N*), and effect allele frequency (*EAF*). Specifically, we calculated the Proportion of Variance Explained (PVE) by each SNP using the standard formulas derived from summary statistics:


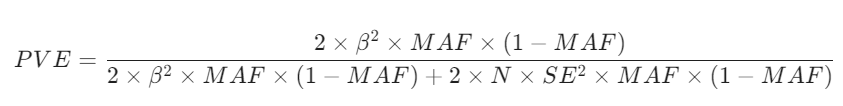


Then, Calculation of F-statistic:


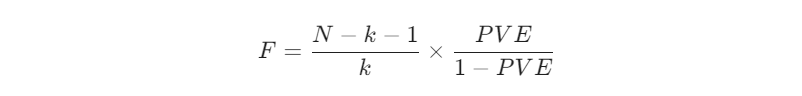


In our study, all selected IVs have F > 29.7, indicating sufficient strength for Mendelian randomization analysis.
